# Insulin receptor responsiveness governs TGFβ‐induced hepatic stellate cell activation: Insulin resistance instigates liver fibrosis

**DOI:** 10.1096/fj.202402169R

**Published:** 2025-03-01

**Authors:** Wang‐Hsin Lee, Evelyn A. Bates, Zachary A. Kipp, Sally N. Pauss, Genesee J. Martinez, Cheavar A. Blair, Terry D. Hinds

**Affiliations:** ^1^ Drug & Disease Discovery D3 Research Center University of Kentucky College of Medicine Lexington Kentucky USA; ^2^ Department of Pharmacology and Nutritional Sciences University of Kentucky College of Medicine Lexington Kentucky USA; ^3^ Markey Cancer Center University of Kentucky Lexington Kentucky USA; ^4^ Department of Physiology University of Kentucky Lexington Kentucky USA; ^5^ Barnstable Brown Diabetes Center University of Kentucky College of Medicine Lexington Kentucky USA

**Keywords:** cirrhosis, diabetes, fatty liver, growth, kinome, MASH, MASLD, PamGene, proliferation

## Abstract

The insulin receptor (INSR) has been shown to be hyperactive in hepatic stellate cells (HSCs) in humans and rodents with liver fibrosis. To explore HSC cellular mechanisms that INSR regulates during pro‐fibrotic stimulation, we used CRISPR‐Cas9 technology. We knocked out a portion of the *INSR* gene in human LX2 HSC cells (*INSR* ^e5‐8^ KO) that regulates insulin responsiveness but not the insulin‐like growth factor (IGF) or transforming growth factor‐β (TGFβ) signaling. The *INSR* ^e5‐8^ KO HSCs had significantly higher cell growth, BrdU incorporation, and lower *TP53* expression that suppresses growth, and they also exhibited increased migration compared to the Scramble control. We treated the scramble control and *INSR* ^e5‐8^ KO HSCs with insulin or TGFβ and profiled hundreds of kinase activities using the PamGene PamStation kinome technology. Our analysis showed that serine/threonine kinase (STK) activities were reduced, and most of the protein‐tyrosine kinase (PTK) activities were increased in the *INSR* ^e5‐8^ KO compared to the Scramble control HSCs. To study gene transcripts altered in activated Scramble control and *INSR* ^e5‐8^ KO HSCs, we treated them with TGFβ for 24 h. We isolated RNA for sequencing and found that the *INSR* ^e5‐8^ KO cells, compared to control HSCs, had altered transcriptional responsiveness to TGFβ stimulation, collagen‐activated signaling, smooth muscle cell differentiation pathways, SMAD protein signaling, collagen metabolic process, integrin‐mediated cell adhesion, and notch signaling. This study demonstrates that reduced INSR responsiveness enhances HSC growth and selectively mediates TGFβ‐induced HSC activation. These findings provide new insights into the development of more effective treatments for liver fibrosis.

## Graphical abstract

Transforming Growth Factor‐β (TGFβ) activated hepatic stellate cells (HSC) induces liver fibrosis. Insulin resistance in the HSCs suppresses *TP53* expression and amplifies proliferation rates and increases αSMA (alpha smooth muscle actin, *ACTA2* gene). This stimulates the proliferation and migration of the HSCs.

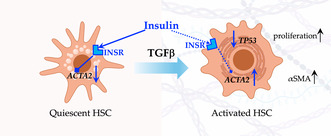

## 1 INTRODUCTION

Nearly one‐third of the world's adult population is predicted to have metabolic dysfunction‐associated steatotic liver disease (MASLD),^1^ a total of over 1.6 billion.^2^ If MASLD is left untreated, it can progress to metabolic‐associated steatohepatitis (MASH) and liver fibrosis^3^; the latter is currently considered irreversible. Hepatic insulin resistance significantly contributes to MASLD.^1^, ^4^ However, in the latter stages of liver disease, the liver sheds lipids that have accumulated in hepatocytes, enhances the growth of hepatic stellate cells (HSCs),^5^ and causes hyperactivity and phosphorylation of the insulin receptor (INSR) in HSCs,^3^ worsening liver dysfunction and commencing cirrhosis.^4^

The HSCs are activated in response to liver damage and are essential for fibrogenesis,^3^, ^5^ contributing up to 80% of collagen I content in hepatic fibrosis.^6^ The liver comprises cell populations that include hepatocytes, HSCs, and other cell types such as Kupffer cells, cholangiocytes, liver sinusoidal endothelial cells, and smooth muscle cells.^7^ The cell types and the proportion of each in the liver vary in response to different nutrients in the diet. A healthy and normal liver comprises around 78% of hepatocytes^8^ and less than 9% of HSCs.^9^ In a healthy liver, HSCs are quiescent (qHSCs), not proliferative, and nonfibrogenic.^10^ When the liver is injured and damage progresses, the qHSCs are activated and proliferate with a reduction of hepatocyte numbers due to apoptosis.^11^ qHSCs can be activated by multiple stimulations, such as transforming growth factor β (TGFβ)^5^ and other cytokines like interleukin‐1β (IL‐1β) and tumor necrosis factor (TNF).^12^ Activated HSCs (aHSCs) proliferate faster than the qHSCs and have upregulated expression of FOXS1,^5^ α‐smooth muscle actin (αSMA), desmin (DES), and collagen type 1 (COL1A1).^6^

We recently showed that the INSR kinase activity was significantly higher in HSCs in men and women with cirrhosis and rodent models of liver fibrosis.^3^ However, the precise mechanism by which insulin receptors regulate HSC activation or proliferation remains unclear. The INSR function is significantly controlled by its tyrosine kinase activity, which is induced by insulin binding to its subunit A domain.^13^ The insulin‐activated INSR signals to downstream proteins such as insulin receptor substrates 1 and 2 (IRS1 and IRS2).^4^ The carboxy‐terminal regions of IRS1 and IRS2 consist of tyrosine phosphorylation sites, where INSR phosphorylates them.^13^ The phosphorylation of IRS1/2 triggers signaling to downstream proteins like the phosphoinositide 3‐kinase (PI3K)/protein kinase B (AKT) pathways that control cellular glucose, fatty acid, and protein metabolism.^4^ The INSR tyrosine kinase is hyperphosphorylated in fibrotic liver disease in humans and rodents in the location of the HSCs, as was shown by visualization of αSMA distribution.^3^ This led us to hypothesize that insulin signaling may mediate HSC proliferation and fibrogenic activities. Therefore, in this study, we reduced insulin's action on INSR signaling function using CRISPR Cas9 to remove an insulin‐responsive portion of the INSR protein (*INSR* ^e5‐8^ KO) in the human HSC cell line, LX2. We used the *INSR* ^e5‐8^ KO and scramble control HSCs to explore the role of INSR in response to TGFβ and insulin stimulations to reveal its role in regulating HSC activation and proliferation.

## 2 MATERIALS AND METHODS

### 2.1 Cell culture

The human hepatic stellate cell (HSC) line LX2 is cultured in DMEM media with 10% FBS and 1% Anti‐Anti at 37°C and 5% CO_2_. For the insulin or IGF2 treatments, the media are changed to DMEM with 10% dialyzed‐FBS and 1% Anti‐Anti. When treated with TGFβ, the treatment is in DMEM either with 10% dialyzed‐FBS and 1% Anti‐Anti for hormone‐free serum media or regular media‐containing sera, 10% FBS and 1% Anti‐Anti.

### 2.2 INSR CRISPR knockout and validation

The human hepatic stellate cell line, LX2, was used to generate the CRISPR knockout of the INSR gene using dual gRNAs expressed by a plasmid from VectorBuilder (Chicago, IL). The vector has a green fluorescent protein (GFP) as a puromycin resistance marker. The plasmid was transfected into the LX2 cells with another plasmid for the expression of Cas9. Twenty‐four hours after transfection, the GFP expression in the cells appeared, followed by puromycin selection and, eventually, the establishment of a stable INSR knockout colony.

To validate the INSR CRISPR knockout in these cells, we did quantitative Real‐Time PCR using primers recognizing the mRNA area within the CRISPR cut site. Then, we validated INSR protein levels through western blot. The total protein was extracted using Mammalian Protein Extraction Reagent (M‐PER) (Thermo Fisher Scientific, Cat. #78501), protease inhibitor cocktail (Sigma P2714‐1BTL), and phosphatase inhibitor cocktail (Fisher PI78420). The total protein extract was quantified using a Microplate BCA Protein Assay Kit (Thermo Scientific #23252). Protein samples were denatured by SDS at 86°C for 8 min and separated by SDS‐PAGE in a Mini‐Protean Stain‐Free gel (Bio‐Rad 4568034 or 4568036). After the electrophoresis, the separated protein was transferred to a polyvinylidene difluoride (PVDF) membrane. Total protein was analyzed using the GelDoc Go imaging system (Bio‐Rad, Hercules, CA, USA). The PVDF membranes were blocked with 3% BSA in TBS at room temperature for 1 h. Following the blocking, the membranes were incubated with primary antibodies against the insulin receptor (Santa Cruz SC‐57342, 1:1000, in TBS) and heat shock protein 90 (HSP90) (R&D AF3776, 1:1000, in TBS) at 4°C overnight. Subsequently, the membranes were incubated with secondary antibodies, anti‐mouse (IRDye 680, red) or anti‐goat (IRDye 800, green), with a 1:10 000 dilution in TBS at 4°C for 2 h, followed by infrared scanning in the Odyssey system (LI‐COR Biosciences, Lincoln, NE, USA). Three biological replicates are presented in the graphs. Densitometry analysis of the images was performed using ImageJ software and normalized with heat shock protein 90 (HSP90) as a control.

### 2.3 Quantitative real‐time PCR analysis

Total RNA was extracted from the cells using a QIAzol Lysis Reagent (Qiagen 79306) and chloroform and then extracted using the RNeasy Mini kit (Qiagen 74106). We measured the total RNA concentrations using the NanoDrop spectrophotometer (Thermo Fisher Scientific, Wilmington, DE). The cDNA was synthesized using the cDNA Reverse Transcription Kit from Applied Biosystems. The cDNA amplification was performed by quantitative real‐time PCR with TrueAmp SYBR Green qPCR SuperMix from Alkali Scientific. The thermocycling setting is 5 min at 95°C, 60 cycles of 15 s at 95°C, 30 s at 60°C, and 30 s at 72°C, finished with a melting curve at temperatures ranging from 60 to 95°C to allow the distinction of specific products. Housekeeping gene expression of 36B4 was used to normalize other gene expressions.

### 2.4 RNA sequencing

The total RNA was purified from LX2 cells using Qiagen Tissue Lyser LT and then extracted using the Qiagen RNeasy kit. The RNA sample concentration was measured using a Thermo Fisher Scientific NanoDrop 2000 spectrophotometer. NovoGene Co performed the RNA sequencing. The Raw fastq files were processed by Kallisto and aligned to the human reference genome GRCh38, which is the latest version. A cutoff of at least 5 fragments per million mapped fragments in at least 75% of the samples was used for filtering genes. Downstream differential gene expressions were analyzed in R with DESeq2. The *p*‐adjusted cutoff was set at <.05 for the significance of differentially expressed genes. Pathway analysis was performed using EnrichR. Genes used as input for EnrichR were calculated using the following equation: [INSRKO (TGFβ‐Veh) − Scramble (TGFβ‐Veh)]. The top and bottom 100 genes were then used as input for EnrichR Gene Ontology analysis.

### 2.5 Proliferation assays

#### 2.5.1 Cell proliferation assay

The *INSR* ^e5‐8^ KO and Scramble HSCs were seeded in 24‐well culture plates (one plate for a time point) at a confluency of 7500 cells per well and were then incubated at 37°C and 5% CO_2_. The TGFβ treatment (5 ng/mL) started on the next day. Every 24 h during the treatment, a plate was treated with 100 μL of MTT (3‐(4,5‐Dimethylthiazol‐2‐yl)‐2,5‐Diphenyltetrazolium Bromide) solution and incubated in the dark within the CO_2_ incubator for 4 h. Afterward, the medium was discarded, and the formazan crystals produced by the cells were solubilized in 300 μL of DMSO. A 100 μL aliquot of this solution was then transferred to a 96‐well plate for absorbance measurement at 570 nm using a Varioskan LUX multiwell plate reader from Thermo Fisher Scientific, USA.

#### 2.5.2 BrdU proliferation assay

A 5‐bromo‐2‐deoxyuridine (BrdU) cell assay was performed to determine proliferation rates. Scramble and *INSR* ^e5‐8^ KO HSCs were seeded in 6‐well plates at 300 000 cells per well. The following day, TGFβ or vehicle treatments were added for 24 h. Immediately following the end of the treatment, the BrdU staining kit from Thermo Fisher Scientific was used as instructed, and flow cytometry was used to measure the results. BrdU was added at 10 μM to the wells and incubated for 30 min, as previously described.^14^ The anti‐BrdU incubation was for 20 min, as previously described.^15^ Flow cytometry then analyzed the samples, and each labeled sample's percentage was graphed.

#### 2.5.3 Cell cycle analysis

The scramble and *INSR* ^e5‐8^ KO HSCs were seeded in DMEM media containing 10% FBS plus 1% Anti‐Anti at 37°C and 5% CO2 overnight. The next day, the cells were treated with 5 ng/mL TGFβ. After a 24‐h treatment, the cells were harvested and washed twice (1500 rpm for 3 min) in wash buffer (PBS + 0.1% bovine serum albumin). 1 × 10^6^ cells were resuspended in 1 mL of wash buffer and placed on ice to cool for 5 min. Then, 3 mL of cold (−20°C) 100% ethanol was added dropwise to each tube while vortexing. The cells were fixed at 4°C overnight. The next day, the cells were washed twice with PBS at 5000 rpm for 5 min. 1 mL of 40 μg/mL propidium iodide staining solution (Sigma P4170) in PBS was added to the cell pellet. Then, 50 μL of 10 μg/mL RNAse A (Thermoscientific EN0531) was added to the tubes and incubated at 37°C for 20 min. The samples were stored at 4°C until ready for flow cytometry analysis.

### 2.6 Migration assay

HSC cells (scramble and *INSR* ^e5‐8^ KO HSCs) were cultured in DMEM media containing 10% dialyzed‐FBS plus 1% Anti‐Anti at 37°C and 5% CO_2_ overnight. After that, 20 000 cells were seeded in each well in a 24‐well transwell plate (VMR PET membrane 8 μm pores, Cat# 10769‐242). The upper‐chamber media was DMEM media with 10% dialyzed‐FBS and 1% Anti‐Anti plus vehicle (10 mM citric acid at pH 3.0) or TGFβ 5 ng/mL. In contrast, the lower chamber media was DMEM with 10% FBS and 1% Anti‐Anti. After cell seeding, we cultured them at 37°C and 5% CO_2_ for 24 h, followed by the cells being fixed by 4% formaldehyde in phosphate buffer saline (PBS) for 30 min. Subsequently, 1% Crystal Violet was added for staining for 1 h. After removing the upper‐chamber cells (not‐migrated cells) using double distilled water and cotton swabs, we counted the lower‐chamber cells (migrated cells) under the microscope.

### 2.7 PamGene PamStation sample preparations

Protein‐tyrosine kinase (PTK) and serine–threonine kinases (STK) PamChips were used to measure kinase activity on the PamStation12 (PamGene International, 's‐Hertogenbosch, The Netherlands). Independent biological replications were run across 3 PamChips for both PTK and STK. The Scramble and *INSR* ^e5‐8^ KO HSCs were treated with 5 ng/mL of TGFβ of 100 nM insulin for 1 h, followed by cell pellet harvesting. The protein was extracted using the Mammalian Extraction Reagent (M‐PER) (Thermo Fischer Scientific, CAT #78503) supplemented with Halt Phosphatase Inhibitor (Thermo Fischer Scientific, CAT #78428) and Protease Inhibitor Cocktail (Sigma, CAT #P2714). The protein concentrations were measured with the Pierce BCA Protein assay kit (Thermo Fischer Scientific, CAT #23225) in triplicate. Protein samples were diluted to a final concentration of 2.5 μg/μL before being added to the PamChips, and 1 μg of protein per sample was used per array for S.T.K. PamChips and 5 μg of protein for P.T.K. PamChips. The kinase phosphorylation activity was quantified by using fluorescently labeled antibodies to detect differential phosphorylation of 196 (for PTK) or 144 (for STK) reporter peptides between different groups and treatment conditions, as previously described.^3^ PamStation12 records relative phosphorylation levels and peptide signal intensities of each phosphor‐peptide sequence every 5 min for 1 h. The exposure times of the CCD camera are 10, 20, 50, and 100 ms. The recorded images were then exported for analysis and kinase mapping.

### 2.8 PamGene PamStation kinome data analysis

The images were analyzed using BioNavigator software (PamGene International, 's‐Hertogenbosch, The Netherlands). Signal ratios were interpreted as fold change (FC) for each phosphor‐peptide average from the triplicates. The minimum threshold values were the same cutoffs as cited in previous literature.^3^, ^16^, ^17^, ^18^ To be considered differential phosphorylation, the thresholds require differential phosphor‐peptide signals (FC) to be ≥1.30 or ≤0.70. Intensity signals produced from linear regression slopes were used in differential analyses—for example, the *INSR* ^e5‐8^ KO TGFβ versus Scramble TGFβ. Phosphor‐peptides with *R* ^2^ < .80 are considered undetectable and nonlinear and are excluded from further analyses. The upstream kinase identification was done as previously described in^19^ by using the Kinome Random Sampling Analyzer (KRSA)^20^ and Upstream Kinase Analysis (UKA)^21^ software packages. MEOW plots (measurements extensively of winner plots) were calculated [Log2 Fold Change (FC) of kinase substrates * Δ confidence (experimental hits/mean hits of 2000 random sampling iterations)] as previously used in Refs. [22, 23] to show individual kinase activity. The kinome phyla tree was produced using CORAL, as described in Ref. [24].

### 2.9 Phyla tree figure generation

Some figures for the kinase tree were created with Adobe Creative Suite, R (4.0.3).^25^ The Human Kinome Paralog Tree Illustration was reproduced courtesy of Cell Signaling Technology, Inc. (www.cellsignal.com).

### 2.10 Western blotting

The cells were cultured to 90% confluency, followed by a one‐hour treatment of TGFβ (Abcam ab50036) at 5 ng/mL, insulin at 100 nM, or IGF2 at 100 ng/mL. The cells were washed with PBS, scraped, and collected for protein extraction. The cell pellets were resuspended with 100 μL M‐PER (ThermoFisher Scientific, Cat no: 78501) with protease inhibitor cocktail (Sigma P2714‐1BTL) and Halt phosphatase inhibitor cocktail (Fisher PI78420). The cell pellets were then homogenized using a pestle for 30 s and incubated for 15 min on ice. Subsequently, the cell lysates were centrifuged at 4°C for 15 min at 45 000 rpm. The supernatant was collected in a new tube, and the protein concentration was measured using a BCA kit (Sigma BCA1). Gel electrophoresis was performed with a Mini‐Protean Stain‐Free gel (Bio‐Rad 4568036), followed by gel imaging using the GelDoc Go imaging system (Bio‐Rad, Hercules, CA, USA). The gel photos were used for total protein measurements. The protein was transferred to PVDF membranes, and the membranes were blocked with 3% BSA or 5% fat‐free milk in TBS at room temperature for 1 h. Subsequently, the membranes were incubated with primary antibodies against pAKT (Cell Signaling 4060, 1:1000, in TBS), AKT (Santa Cruz SC‐1619, 1:1000, in TBS), pERK (Cell Signaling 9106, 1:1000, in TBS), and ERK (Cell Signaling 9102, 1:1000 in TBS) at 4°C overnight. After three washes in TBS with 0.1% Tween 20, the membranes were incubated with a secondary antibody of anti‐rabbit (IRDye 680, red), anti‐mouse (IRDye 680, red), or anti‐goat (IRDye 800, green) labeled with IRDye infrared dye (LI‐COR Biosciences) (1:10 000 dilution in TBS) for two hours at 4°C.^26^ The blots were visualized by infrared scanning in the Odyssey system (LI‐COR Biosciences, Lincoln, NE, USA), and the densitometry was performed using ImageJ.

### 2.11 Imaging

Scramble LX2 and *INSR* ^e5‐8^ KO LX2 cells were seeded on glass coverslips in 6‐well plates in DMEM media containing 10% FBS plus 1% Anti‐Anti at 37°C and 5% CO_2_ overnight. We seeded 35 000 cells in each well, followed by a 24‐h vehicle (10 mM Citric Acid, pH 3.0) or TGFβ (Abcam, ab50036) treatment. After the treatment, the cells were fixed with 4% formaldehyde in PBS. The fixed cells were washed with PBS and then permeabilized with 0.5% Triton X‐100 at room temperature for 10 min. The permeabilized cells were washed with PBS again and then blocked with 5% BSA in PBS at room temperature for 1 h. Following the blocking, the cells were incubated with the primary antibody for αSMA (Cell Signaling, #19245) at a 1:200 dilution in 5% BSA at 4°C overnight. After primary antibody incubation, the cells were washed with PBS and incubated with Alexa Fluor 594 anti‐Rabbit IgG (H + L) secondary antibody (Invitrogen, #A21207) at a 1:200 dilution and DAPI (MilliporeSigma, #28718‐90‐3) at a 0.5 μg/mL concentration in 5% BSA at room temperature for 1 h. Subsequently, the cells were washed with PBS again and made into slide samples with mounting media (Invitrogen, #P36961). Immunofluorescent imaging was performed using the Olympus 1X81 inverted fluorescent microscope system with the same exposure setting and amplification. The image densitometry was performed with ImageJ.

### 2.12 Statistics

All data is presented as mean ± SEM. A Student *t*‐test or one‐way analysis of variance with a post hoc test (Dunnett's) was used for analyzing differences between groups, and *p* < .05 is considered significant. Statistical analysis and graph making were performed using GraphPad Prism9 (GraphPad Software, Inc., San Diego, CA). All raw data from the PamStation was processed and analyzed using BioNavigator, which provided the data for individual kinases. Kinase data from the PamStation was also analyzed by the kinase random sampling analysis package (KRSA) (resource link: https://github.com/CogDisResLab/KRSA) in R (version 4.1.2), which is compatible with both individual kinases and kinase families. Results from both pipelines were used and presented.

## 3 RESULTS

### 3.1 CRISPR INSR knockout in human HSCs and the effects on INSR gene expression, cell growth, and migration

**FIGURE 1:**
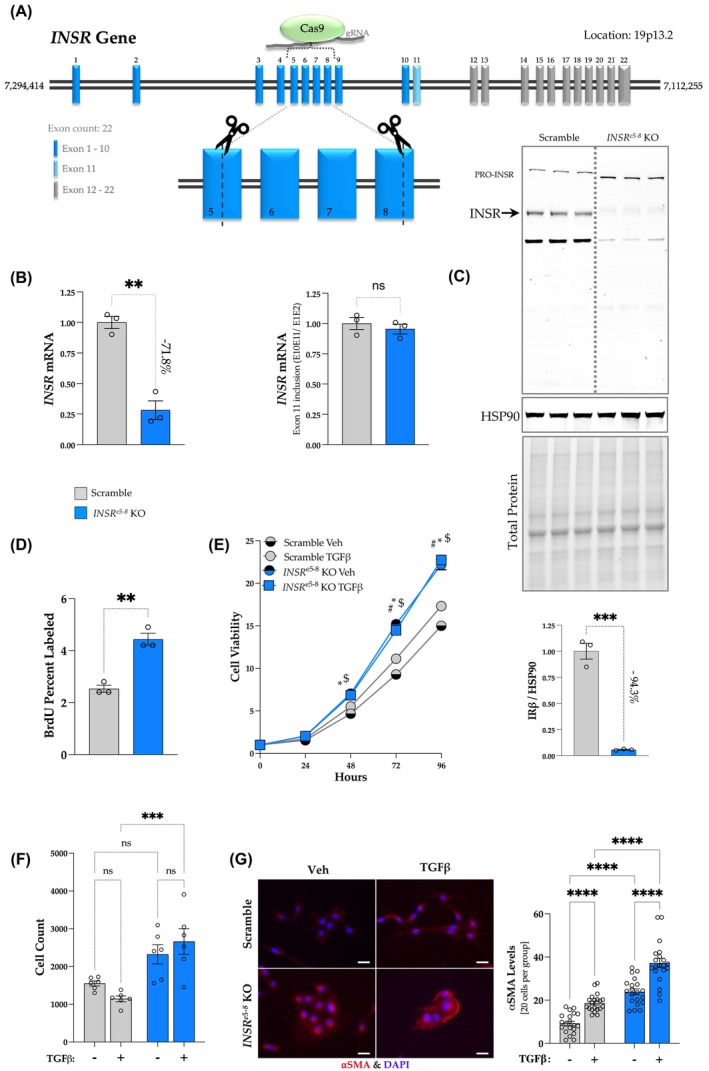
Generation of CRISPR against *INSR* gene in human hepatic stellate cells and validation. (A) Diagram of the CRISPR knockout of *INSR* gene. (B) Real‐time PCR results show the validation of the *INSR* gene KO on *INSR* mRNA alternative splicing (*p  <.05; **p < .01; ***p < .001; *n* = 3 each; unpaired *T*‐test; ±SEM), and (C) Western Blotting showing the protein expression of the insulin receptor (INSR) and heat shock protein 90 (HSP90) as a control in Scramble and *INSR* ^e5‐8^ KO HSCs (*p < .05; **p  <.01; ***p < .001; *n* = 3 each; unpaired *T*‐test; ±SEM). (D) BrdU incorporation in the scramble and *INSR* ^e5‐8^ KO HSCs. (E) Cell growth assay results of scramble and *INSR* ^e5‐8^ KO HSCs treated with tumor growth factor‐β (TGFβ) 5 ng/mL for 0 to 96 h. *p < .05 in INSR KO Veh v.s. Scramble Veh; $*p* < .05 in INSR KO TGFb v.s. Scramble TGFb; #*p* < .05 in Scramble TGFb v.s. Scramble Veh (*n* = 6). (F) Cell migration assay results of scramble and *INSR* ^e5‐8^ KO HSCs treated with TGFβ 5 ng/mL for 24 h. (G) Immunostaining with α‐smooth muscle actin (αSMA) antibody and DAPI for nucleus visualization in scramble and *INSR* ^e5‐8^ KO HSCs treated with TGFβ 5 ng/mL for 24 h (*p < .05; **p  <.01; ***p < .001, *n* = 3 each; two‐way ANOVA; ± SEM).

We used CRISPR Cas9 to remove a fragment of the *INSR* gene from the middle of exon 5 to almost the end of exon 8, where these and exons 6 and 7 are completely removed to generate the *INSR* ^e5‐8^ KO with amino acids 395–605 removed compared to intact INSR protein in Scramble control cells (Figure 1A). This reduces *INSR* mRNA expression by 71.8% (*p* = .0014) (Figure 1B, left) and functional INSR protein subunit β by 94.3% (*p* = .0002) (Figure 1C). Since *INSR* mRNA alternative splicing at exon 11 regulates insulin receptor function, we also measured the exon 11 inclusion to see the effects of the *INSR* ^e5‐8^ KO on mRNA alternative splicing, and no differences were observed (*p* = .5119) (Figure 1B, right). To determine whether INSR controls HSC cell growth, we performed a BrdU incorporation assay to measure proliferation rates. The *INSR* ^e5‐8^ KO, compared to Scramble HSCs, had significantly higher BrdU incorporation, indicating higher proliferation (Figure 1D). Next, to further explore the growth rates in the *INSR* ^e5‐8^ KO and Scramble HSCs, we performed the MTT (3‐(4,5‐Dimethylthiazol‐2‐yl)‐2,5‐Diphenyltetrazolium Bromide) growth assay with or without TGFβ stimulation over 96 h. The MTT assay showed that *INSR* ^e5‐8^ KO HSCs presented higher cell viability when treated with vehicle or TGFβ for 48 to 96 h, compared with Scramble control with the same treatment and time (*p* = .0011 for comparison of TGFβ treatment at 48 h; *p* < .0001 for all the other comparisons) (Figure 1E). The TGFβ increased Scramble HSC viability at 48–96 h of stimulation (*p* < .0001 for comparisons at 48, 72, and 96 h). However, the TGFβ treatment did not increase *INSR* ^e5‐8^ KO HSC cell growth compared to the vehicle‐treated group. Migration ability is an essential function of HSC activation. To determine whether the *INSR* ^e5‐8^ KO and Scramble HSCs had changes in migrating capability in response to a 24‐h stimulation, we performed a migration assay using PET‐membrane transwell plates with 8 μm pores. The result showed that reducing the responsiveness of INSR in the *INSR* ^e5‐8^ KO significantly increased HSC cell migration with the TGFβ treatments, compared to the Scramble HSCs with the same treatments (*p* = .0004) (Figure 1F). However, TGFβ stimulation did not change migration in *INSR* ^e5‐8^ KO HSCs. Lastly, to determine the phenotypic responses of activated HSCs, we treated the Scramble or *INSR* ^e5‐8^ KO HSCs with TGFβ or vehicle for 24 h and did immunostaining for αSMA. The TGFβ treatments significantly increased αSMA levels in Scramble and *INSR* ^e5‐8^ KO HSCs (Figure 1G). However, the *INSR* ^e5‐8^ KO HSCs had significantly higher αSMA than Scramble in the vehicle and TGFβ‐treated groups. Another observation is that the TGFβ treatments in the Scramble made the cell appearance longer and more fibrotic‐like, but this was not observed in the *INSR* ^e5‐8^ KO HSCs. The increased proliferation rates observed with higher proliferation assays were associated with the reduced *TP53* mRNA expression measured in the TGFβ‐treated Scramble control (Figure S1A). The *TP53* mRNA expression was significantly reduced in the *INSR* ^e5‐8^ KO HSCs without TGFβ treatments, and it remained near the same level with the treatments. The *TP53* gene encodes the protein for tumor suppressor p53, which inhibits HSC growth and fibrogenesis and activates apoptosis when DNA damage is detected.^27^, ^28^ The function of p53 is to promote HSC senescence^28^ and suppress hepatic cancer upon liver damage.^29^ We also measured the cell cycle in the *INSR* ^e5‐8^ KO and Scramble HSCs, and G1 was suppressed by TGFβ treatments in both groups (Figure S1B). However, the TGFβ‐induced G2 phase was only observed in the Scramble and not in the *INSR* ^e5‐8^ KO HSCs, likely leading to escalated mitosis and growth in these cells. These findings indicate the important role of INSR in regulating HSC proliferation under pro‐fibrogenic activation and stimulation.

### 3.2 Insulin signaling pathway response to INSR ligands and TGFβ treatment in INSR^e5‐8^ KO and scramble HSCs

**FIGURE 2:**
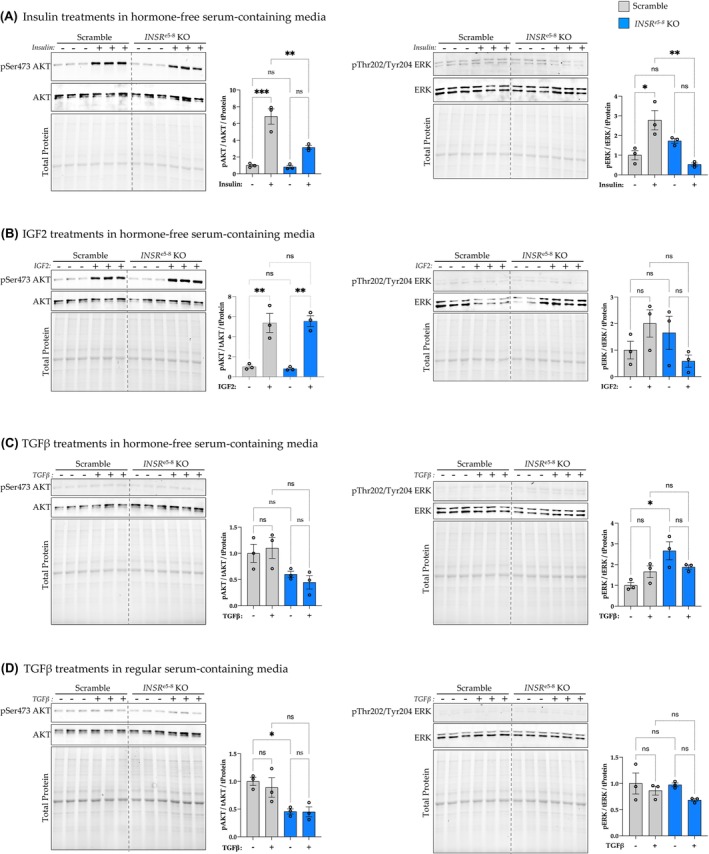
AKT and ERK are differentially phosphorylated in *INSR* ^e5‐8^ KO and control HSCs in response to insulin, IGF2, or TGFβ. Western blot results showing phosphorylated pAKT and pERK and total protein expressions in *INSR* ^e5‐8^ KO HSCs treated for 1 h with (A) insulin 100 nM, (B) insulin‐like growth factor‐2 (IGF2) 100 ng/mL, (C) transforming growth factor‐β (TGFβ) 5 ng/mL in hormone‐free serum media, or (D) TGFβ 5 ng/mL in normal serum‐containing media (*p < .05; **p < .01; ***p  <.001, *n* = 3 each; two‐way ANOVA; ±SEM). ns = not significant

We also wanted to determine if the *INSR* ^e5‐8^ KO HSCs have altered insulin signaling. Thus, we cultured the scramble and *INSR* ^e5‐8^ KO HSCs with vehicle or insulin for 1 hr and did an immunoblotting probing for pAKT and phosphorylated extracellular signal‐regulated kinase (pERK), the two essential hubs in the insulin signaling pathway (Figure 2A,B). We found that *INSR* ^e5‐8^ KO decreased pAKT and pERK expressions when treated with insulin, as we expected. However, the insulin treatment still increased AKT phosphorylation in *INSR* ^e5‐8^ KO HSCs, compared with *INSR* ^e5‐8^ KO HSCs treated with a vehicle (Figure 2A, left). On the other hand, *INSR* ^e5‐8^ KO HSCs had significantly decreased insulin's increasing effect on pERK expression (Figure 2A, right). Besides insulin, insulin‐like growth factor 2 (IGF2) can affect insulin signaling similarly. Therefore, we did immunoblotting probing for pAKT and pERK in *INSR* ^e5‐8^ KO HSCs treated with IGF2 for 1 h to see if they are differentially regulated. We found that IGF2 treatment increased AKT phosphorylation but not pERK expression in both scramble and *INSR* ^e5‐8^ KO HSCs, compared to the same cells with vehicle treatments (Figure 2B). The *INSR* ^e5‐8^ KO HSCs did not change IGF2's effects on pAKT and pERK. We also measured the pAKT and pERK expression in scramble and *INSR* ^e5‐8^ KO HSCs treated with TGFβ in both hormone‐free and regular media (Figure 2C,D). The TGFβ treatments did not change pAKT and pERK expressions in scramble and *INSR* ^e5‐8^ KO HSCs.

### 3.3 Determination of INSR‐dependent gene networks

**FIGURE 3:**
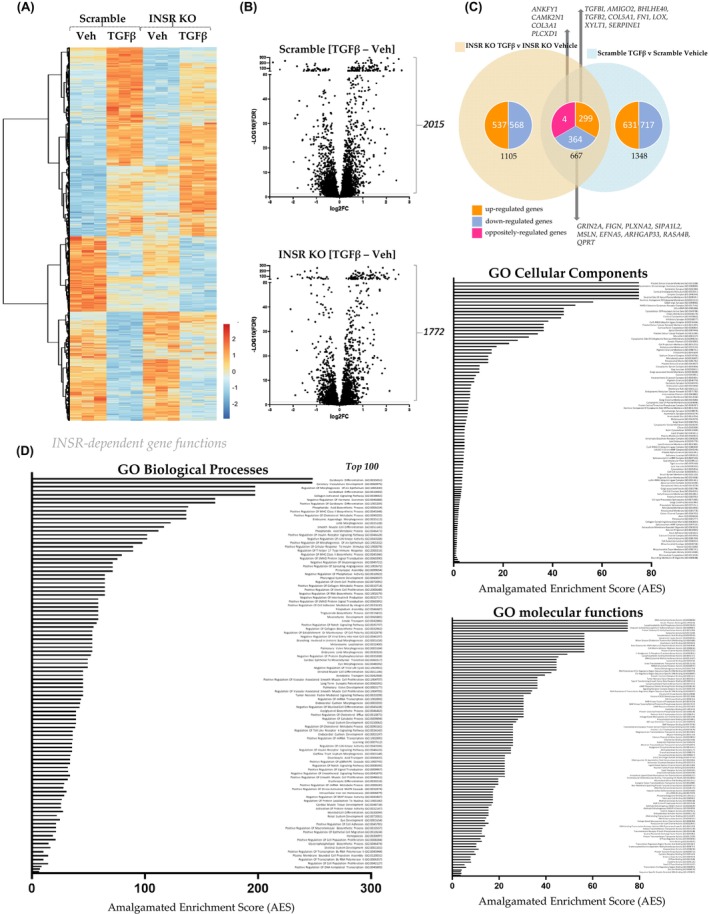
RNA‐sequencing of TGFβ‐induced pathways in scramble and *INSR* ^e5‐8^ KO HSCs. The *INSR* ^e5‐8^ KO and Scramble control HSCs were treated with 5 ng/μL TGFβ or vehicle for 24 h, and RNA was extracted for RNA sequencing. (A) Heatmap analysis of RNA‐seq data. (B) Volcano plots of differentially expressed genes (DEGs) in the scramble and *INSR* ^e5‐8^ KO HSCs treated with vehicle or TGFβ. (C) Venn diagram showing the number of genes changed in the *INSR* ^e5‐8^ KO HSCs TGFβ versus Veh and Scramble control TGFβ versus Veh. The genes shared between the comparisons are shown in the middle, separated by up, down, and oppositely regulated genes. (D) The Gene Ontology (GO) pathway analysis for INSR‐dependent genes. The analyses are divided into biological, cellular, and molecular functions. The amalgamated enrichment score (AES) is a combination of the GO score and the significance value of each pathway.

To establish the gene networks controlled by INSR function, we did RNA sequencing of the scramble and *INSR* ^e5‐8^ KO HSCs treated with vehicle and TGFβ 5 ng/μL for 24 h. We created a heatmap for visualizing the gene expression profile using the normalized expression values for the measured genes (Figure 3A). The heatmap shows the differentially altered gene expression profile, suggesting sets of genes are mediated by differentially regulated in the *INSR* ^e5‐8^ KO HSCs by TGFβ treatment. To examine DEGs that are under the control of INSR and TGFβ in HSCs, we visualized them with volcano plots of scramble [TGFβ‐Veh] and *INSR* ^e5‐8^ KO HSCs [TGFβ‐Veh] (Figure 3B). The scramble LX2 has 2015 DEGs, whereas the *INSR* ^e5‐8^ KO HSCs have 1772 DEGs, indicating that the loss of INSR induced a 243‐DEG difference. To better show the genes regulated by INSR, we made a Venn Diagram by using the gene list with a cutoff of *p*‐adjusted <.05 and TGFβ groups subtracted by the vehicle [TGFβ‐Veh] (Figure 3C). There were 667 shared genes that changed between the two comparisons, representing the INSR‐dependent genes. Of those genes, 299 were increased, and 364 were decreased in both comparisons. We highlighted the top 10 up‐regulated genes (*TGFBI, AMIGO2, BHLHE40, TGFB2, COL5A1, FN1, LOX, XYLT1, SERPINE1*) and the top 10 down‐regulated genes (*GRIN2A, FIGN, PLXNA2, SIPA1L2, MSLN, EFNA5, ARHGAP33, RASA4B, QPRT*). Of interest in our analysis are the 4 oppositely regulated genes, which include *ANKFY1, CAMK2N1, COL3A1*, and *PLCXD1*. Based on INSR‐dependent DEG data, we created a Gene Ontology (GO) pathway analysis with three categories: biological, cellular, and molecular functions (Figure 3D). We performed the amalgamated enrichment score (AES) to score the pathways, as we have previously described,^30^ which is a combination of the number of genes involved in a pathway and the significance of the change of the pathway. In the GO Biological Functions associated with INSR controls, we found that fibrosis‐associated pathways like collagen‐activated signaling and smooth muscle cell differentiation are in the top 15 altered pathways. In addition, it also shows that the pathways associated with INSR signaling, cellular response to insulin stimulation, SMAD protein signaling, collagen metabolic process, integrin‐mediated cell adhesion, and notch signaling are significantly changed (Figure 3D, left). As for the GO Cellular Components analysis, we found that cellular components involved in keratin filament, platelet functions, actin cytoskeleton, and lipid droplets are significantly altered (Figure 3D, top right). A deeper investigation using the GO Molecular Function analysis shows that many fibrosis‐related functions are changed, including keratin filament binding, cell‐matrix adhesion mediator activity, TGFβ receptor II binding, and cyclic adenosine monophosphate response element binding protein (CBP) binding. Interestingly, some metabolic functions are also altered, such as lipoprotein lipase activity and fatty‐acyl CoA binding (Figure 3D, bottom right).

**FIGURE 4:**
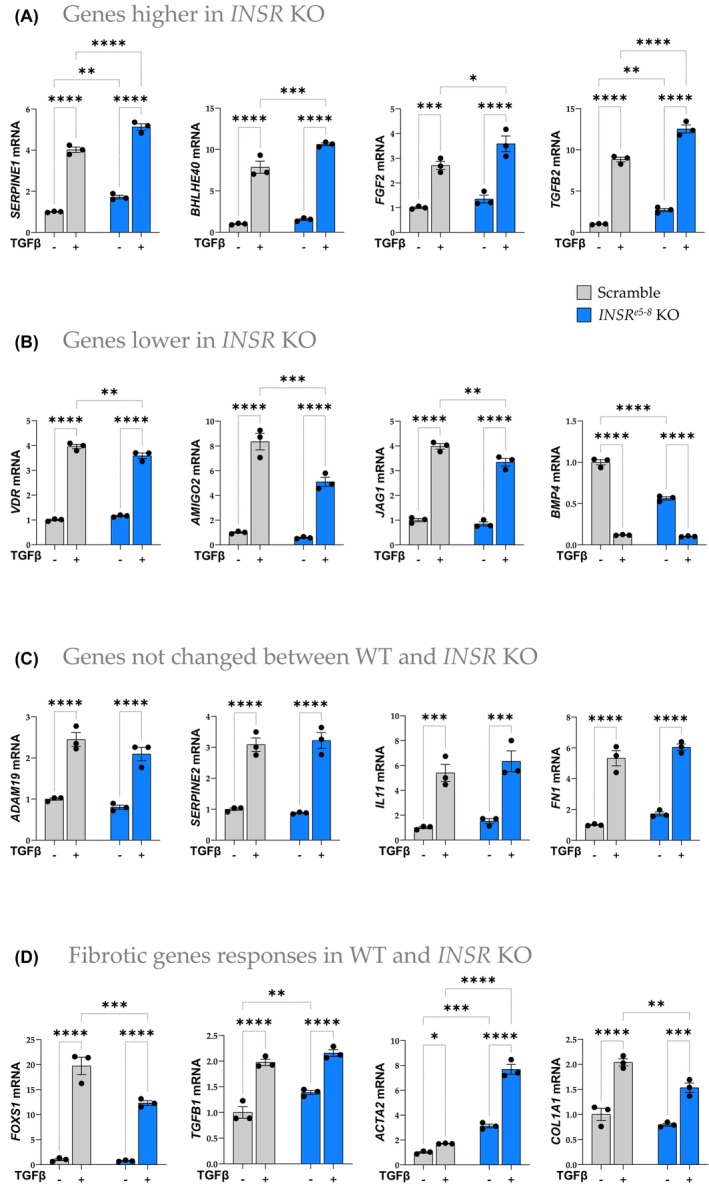
Real‐time PCR validation of the RNA‐sequencing data. Real‐time PCR results to validate gene expressions that were (A) increased, (B) decreased, (C) not changed, or (D) fibrotic gene responses for the RNA‐sequencing results in *INSR* ^e5‐8^ KO and Scramble HSCs treated with TGFβ 5 ng/mL for 24 h (*p < .05; **p < .01; ***p < .001, *n* = 3 each; two‐way ANOVA; ±SEM).

To validate the identified genes in RNAseq results (Figure 3), we measured some by real‐time PCR. We showed genes that increased (Figure 4A), decreased (Figure 4B), and did not change (Figure 4C) in *INSR* ^e5‐8^ KO HSCs treated with TGFβ, compared to its scramble control HSC counterpart. In addition, we measured fibrosis‐associated genes and validated the efficacy of TGFβ treatment (Figure 4D). We found that the loss of INSR significantly changed HSC response to TGFβ treatment. This indicates that INSR is crucial in HSC cellular reaction to a fibrogenic environment. Thus, we used the PamGene kinome technology to uncover the real‐time kinase activities in cellular signaling pathways.

### 3.4 Determination of INSR‐dependent kinase pathways in human hepatic stellate cells

The RNAseq data addressed the transcriptional profile in *INSR* ^e5‐8^ KO and scramble control HSCs in response to TGFβ. This triggered our curiosity to explore the kinase networks and pathways INSR regulates in a pro‐fibrogenic environment. Thus, we measured the kinase activities with the PamGene PamStation technology. We quantified kinase activities using this technology by detecting the sample‐induced phosphorylation of 144 serine–threonine kinase peptide substrates on the STK chip and 196 tyrosine kinase peptide substrates on the PTK chip. The scramble control and *INSR* ^e5‐8^ KO HSCs were treated with vehicle or 5 ng/mL TGFβ or 100 nM insulin for 1 h, and the protein lysate was extracted to measure kinase activity.

**FIGURE 5:**
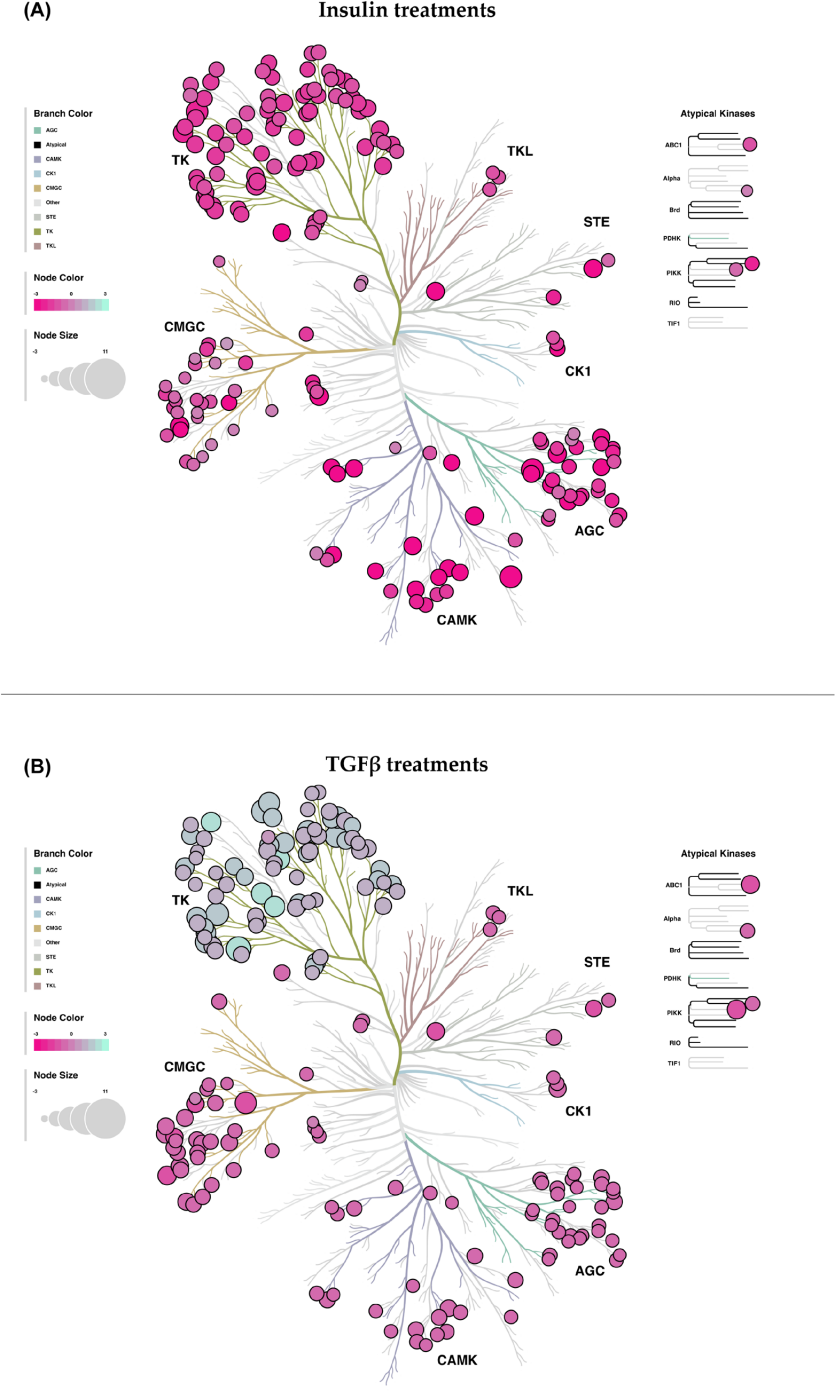
Paralogous phylogenic correlations between differentially activated kinases in response to insulin or TGFβ treatments. The *INSR* ^e5‐8^ KO and scramble HSCs treated with (A) insulin or (B) TGFβ treatments were compared using the PTK and STK data for analyses. Node color represents the median kinase statistic, and node size refers to the mean final kinase score on the bubble plot with the paralogous phylogenetic trees.

**FIGURE 6:**
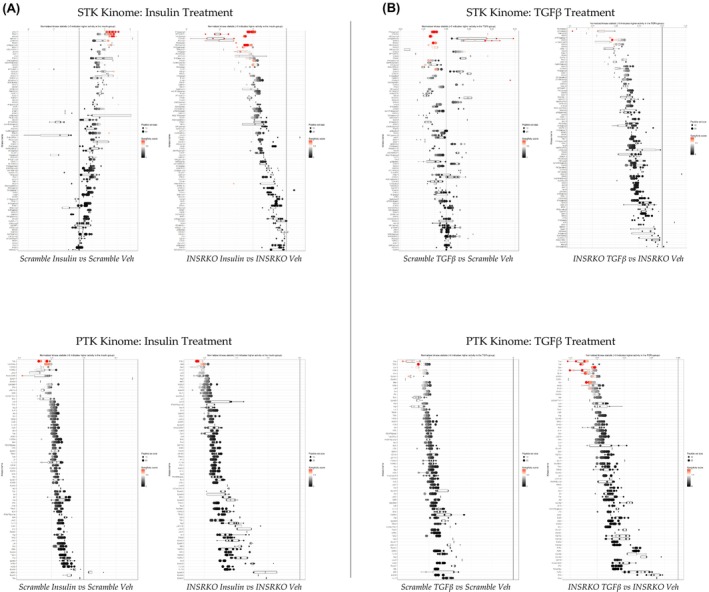
Individual protein‐tyrosine kinase (PTK) and serine–threonine kinase (STK) activities in response to insulin or TGFβ stimulation Individual protein‐tyrosine and serine–threonine kinases plotted using normalized kinase statistics showing hyperactive and hypoactive kinases in *INSR* ^e5‐8^ KO and scramble control HSCs. (A) Comparisons between *INSR* ^e5‐8^ KO and scramble HSCs treated with insulin. (B) Comparisons between *INSR* ^e5‐8^ KO and scramble HSCs treated with TGFβ.

We presented the data with a kinase phyla tree diagram to visually demonstrate kinase activity with the phosphorylation condition among the measured substrates (Figure 5). The size of the circles indicates the kinase mean final score. The color of the circles refers to the median kinase statistic. These values represent the stimulated kinase activity in the *INSR* ^e5‐8^ KO compared to scramble HSCs in response to TGFβ or insulin. The results showed that the loss of INSR decreases tyrosine kinase activity in response to insulin treatment (Figure 5A) and increases tyrosine kinase activity in response to TGFβ treatment (Figure 5B). We also compare the kinase activity changes in STK and PTK in *INSR* ^e5‐8^ KO and scramble HSCs in response to treatments (Figure 6). We found that insulin treatment increases STK activity in scramble control HSCs (Figure 6A, top left), and losing INSR reduces STK activity in response to insulin treatment in HSCs (Figure 6A, top right). This effect is not observed in PTK activity, and insulin treatment generally reduces PTK activity in scramble control and *INSR* ^e5‐8^ KO HSCs (Figure 6A, bottom left and right). The magnitude of PTK suppression was greater in the *INSR* ^e5‐8^ KO HSCs, near 1.5 compared to 0.3 for Scramble HSCs. INSR is membrane‐bound and interacts with multiple other receptors and signaling proteins.^4^ The data indicate that exons 5–8 of the *INSR* gene disruption likely mediate other binding partner effects, as shown in the STK and PTK data. Mutations in this region strongly impair ERK phosphorylation but induce wild‐type levels of IRS‐1 phosphorylation.^31^, ^32^

Once insulin binds, INSR induces a protein conformational change in this region,^33^ which is likely important for other binding partners.

As for TGFβ treatment, we found they simultaneously decreased and increased some of the STK activity in Scramble control HSCs (Figure 6B, top left). Losing INSR reduces STK activity in response to TGFβ stimulation in HSCs (Figure 6B, top right). The *INSR* ^e5‐8^ KO HSCs had changes in STK activity patterns in response to insulin and TGFβ treatments (Figure 6A, top right, and Figure 6B, top right). In addition, similar to insulin treatment comparisons, the *INSR* ^e5‐8^ KO HSCs did not change the PTK responsiveness to TGFβ stimulation (Figure 6B, bottom left and right).

**FIGURE 7:**
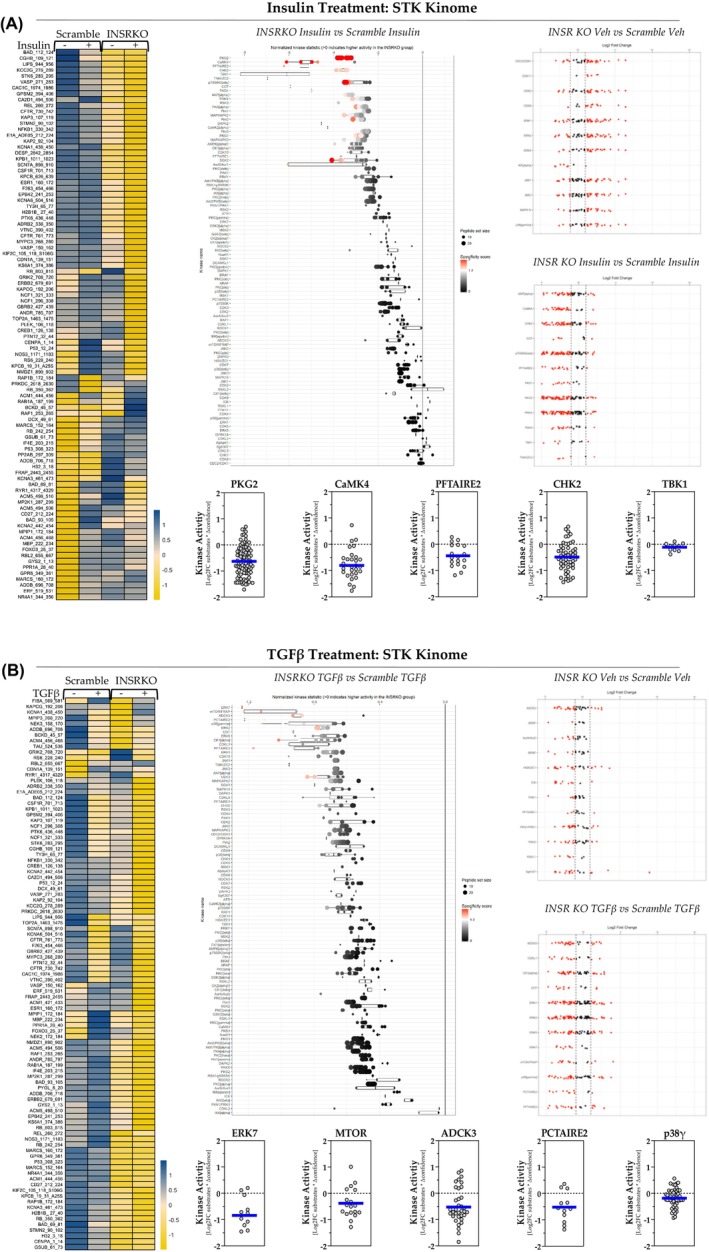
Serine–threonine kinase (STK) activity in response to insulin or TGFβ. (A, B) Heatmap analysis of differentially phosphorylated serine–threonine kinases substrates (A, left and B, left). The serine–threonine kinases plotted by normalized kinase statistics show hyperactive and hypoactive kinases in *INSR* ^e5‐8^ KO and scramble HSCs in response treatments (A, middle and B, middle). Individual serine–threonine kinases were plotted by the log2 fold change of their substrates (A, right and B, right). MEOW plots show the individual kinase activities (A, bottom, and B, bottom). The blue line in MEOW plots indicates the average activity among all the included substrates.

When comparing *INSR* ^e5‐8^ KO HSCs with Scramble HSCs for insulin treatment, we found that the loss of INSR decreased the STK substrate phosphorylation, as shown in the heat map (Figure 7A, left). We performed an upstream kinase analysis to show individual kinase activities and found a decrease in STK activity (Figure 7A, middle). To find the most changed STKs, we plotted the data using log2 fold change (Figure 7A, right). We identified the top 12 changed STKs in *INSR* ^e5‐8^ KO HSCs in response to only vehicle treatment (Figure 7A, top right) or insulin treatment alone (Figure 7A, middle right). Among the most changed kinases in insulin treatment, *INSR* ^e5‐8^ KO HSCs had reduced kinase activity of protein kinase G (PKG), calcium/calmodulin‐dependent protein kinase type IV (CaMK4), cyclin‐dependent kinase 15 (PFTAIRE2), checkpoint kinase 2 (CHK2), and TANK‐binding kinase 1 (TBK1) by 62.7%, 80.3%, 43.9%, 48.3%, and 10.9%, respectively (indicated by the blue lines in MEOW plots) (Figure 7A, bottom figures). We did the same analysis for TGFβ treatment groups (Figure 7B). We found that the *INSR* ^e5‐8^ KO HSCs had decreased HSC's STK activities in TGFβ treatment (Figure 7B, middle). We identified that ERK7, mammalian target of rapamycin (MTOR), aarF‐domain‐containing kinase 3 (ADCK3), cyclin‐dependent kinase 17 (PCTAIRE2), and p38γ are in the top 12 hypoactive STKs in the comparison between only TGFβ groups (Figure 7B, bottom figures). In these results, ERK7, PCTAIRE2, p38γ, ADCK3, and MTOR activities are reduced by 84.3%, 51.9%, 18.2%, 52.4%, and 37.9%, respectively.

**FIGURE 8:**
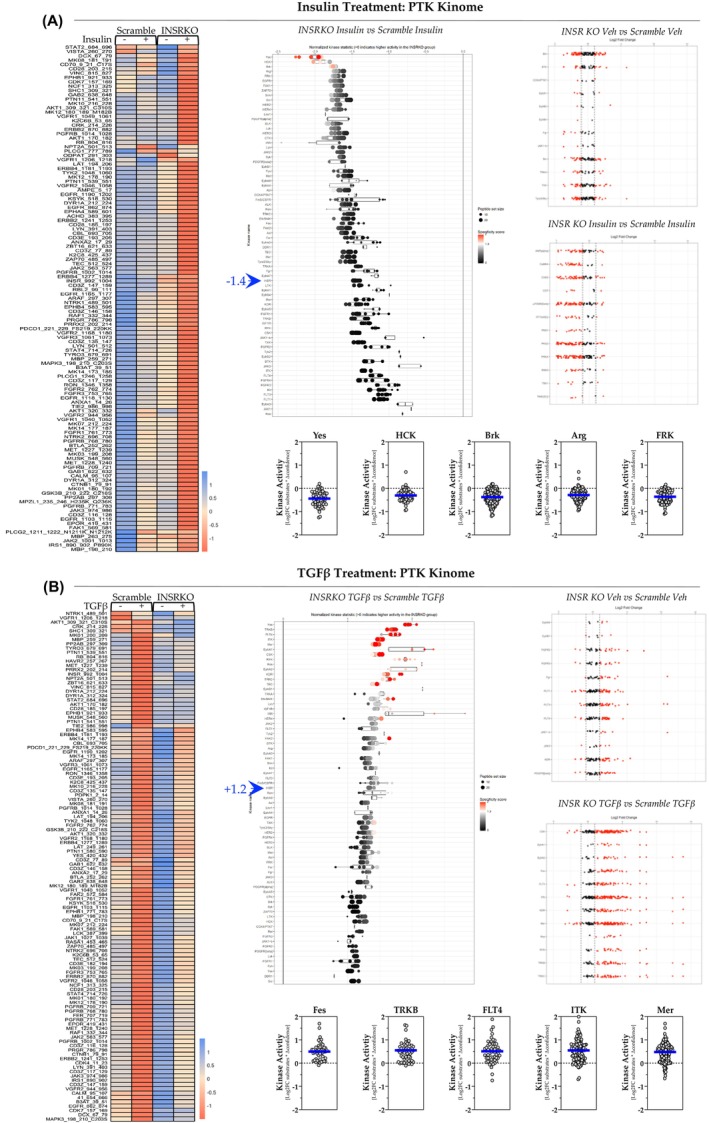
Protein‐tyrosine kinase (PTK) activity in response to insulin or TGFβ. (A, B) Heatmap analysis of differentially phosphorylated protein‐tyrosine kinase substrates (A, left and B, left). Individual protein‐tyrosine kinase plotted by normalized kinase statistics showing hyperactive and hypoactive kinases in *INSR* ^e5‐8^ KO and scramble HSCs in response treatments (A, middle and B, middle). The blue arrows indicate the activity shift of INSR when treated with insulin or TGFβ. Individual protein‐tyrosine kinases were plotted by the log2 fold change of their substrates (A, right and B, right). MEOW plots show the individual kinase activities (A, bottom, and B, bottom). The blue line in MEOW plots indicates the average activity among all the included substrates.

The same analysis was also performed for PTK data (Figure 8A,B). We found that the loss of INSR regulates HSC's PTK responsiveness to insulin and TGFβ oppositely, reducing PTK activity in insulin treatment (Figure 8A, middle) and increasing PTK activity in TGFβ treatment (Figure 8B, middle). Individual kinase activities were selected from the most changed kinases in insulin and TGFβ treatment and are shown as MEOW plots (Figure 8A, bottom figures, and 8B, bottom figures). Among the most changed kinases in insulin treatment, the *INSR* ^e5‐8^ KO HSCs had reduced kinase activity of YES Proto‐Oncogene 1 (YES), hematopoietic cell kinase (HCK), Breast tumor kinase (BRK), Abl‐related gene (ARG), and Fyn‐related kinase (FRK) by 44.0%, 30.2%, 37.5%, 28.5%, and 35.7%, respectively (Figure 8A, bottom figures). In TGFβ treatment, the *INSR* ^e5‐8^ KO HSCs had increased the kinase activities of proto‐oncogene c‐Fes/Fps (FES), tropomyosin receptor kinase B (TRKB), fms‐related tyrosine kinase 4 (FLT4), IL2 Inducible T Cell Kinase (ITK), and MER receptor tyrosine kinase (MER) by 49.6%, 55.0%, 51.0%, 54.6%, and 47.9%, respectively (Figure 8B, bottom figures). These findings also show that the *INSR* ^e5‐8^ KO HSCs had altered STK (Figure 7B) and similar PTK (Figure 8B) responses with TGFβ treatments.

In summary, our findings indicate that INSR plays an essential role in HSCs' proliferation, migration, pathway activations, and kinase activities in response to TGFβ induced fibrogenic environment. The affected pathways include fibrosis‐related functions, insulin signaling, TGFβ signaling, and collagen metabolism.

## 4 DISCUSSION

The investigations here demonstrate the involvement of INSR crosstalk in TGFβ and insulin signaling in human HSCs. Although it has been shown that INSR and insulin signaling are involved in HSC response to a fibrotic environment,^34^ the pathways regulating HSC responses have not been fully unveiled until this work. Our study demonstrated that INSR has an involvement in regulating TGFβ‐induced HSC activation by regulating kinase activities and gene expression in fibrogenesis‐related signaling pathways. The loss of INSR reduced HSC's gene transcriptional activity to TGFβ by 243 genes, indicating a specific set of genes regulated by TGFβ‐INSR. There were also obvious changes in kinase pathways in the *INSR* ^e5‐8^ KO compared to control HSCs. These findings suggest that INSR may be involved in liver fibrosis; contrarily, MASLD is a known hepatic insulin‐resistant state manifested from metabolic dysfunction.^4^ However, the function of INSR in the latter stages of liver disease might be dichotomous.

Our previous study presented a hepatic kinome atlas in cirrhotic human liver and hepatic fibrosis rodent models using PamGene technology and found both had INSR hyperactivity.^3^ Surprisingly, the distribution of phosphorylation of INSR overlaps with αSMA, a product and marker of aHSCs. This finding and our study indicate that INSR signaling is involved in HSC function. However, the role of INSR in regulating HSC fibrogenesis activity is debated. Some studies found that activating INSR tyrosine kinase signaling promotes HSC fibrogenesis.^34^, ^35^ Another study advocated that reduced INSR signaling increased the activation of HSCs.^36^ Our study showed that losing intact INSR leads to higher *TGFB1* and *ACTA2* (αSMA) expression associated with higher fibrogenesis activities.^6^, ^37^ In addition, we found that the reduced responsiveness of INSR increased HSC proliferation, as measured by BrdU incorporation and MTT growth assays. Although TGFβ treatment did not significantly alter cell growth in *INSR* ^e5‐8^ KO HSCs, it enhanced cell growth in Scramble control HSCs with intact INSR protein. The loss of insulin‐INSR‐induced kinase signaling may have a growth‐promoting effect in HSCs similar to that of TGFβ stimulation, as TGFβ did not increase the growth rate in the *INSR* ^e5‐8^ KO HSCs. These findings could be related to the significantly reduced p53 expression observed in the *INSR* ^e5‐8^ KO HSCs, which has been shown to induce HSC senescence and inhibit growth and fibrogenesis.^27^, ^28^ These findings indicate that intact INSR can inhibit HSC proliferation.

Our study demonstrated that kinase activity in real‐time changes in TGFβ‐activated HSCs when insulin‐INSR signaling is reduced. Using the advanced PamGene PamStation technology, we found the kinases that may play critical roles in the *INSR* ^e5‐8^ KO HSCs compared to scramble control for functional changes. This study also presented evidence that PTKs and STKs are changed in response to TGFβ stimulation, and this was not observed in the *INSR* ^e5‐8^ KO HSCs. We found PTKs tend to be hyperactive and STKs to be hypoactive. PTKs frequently initiate downstream signals upon activation.^38^ INSR is a transmembrane PTK with tyrosine kinase activity located on its intracellular β subunits. When its extracellular α subunits bind to ligands such as insulin, the tyrosine kinases on the β subunits undergo autophosphorylation. This autophosphorylation event subsequently phosphorylates downstream substrates, such as IRS1, thereby initiating the INSR signaling pathway in the cell. The increased activity of PTKs may indicate increased receptor activation events. In response to insulin treatment, the *INSR* ^e5‐8^ KO HSCs had decreased PTK signaling for YES, HCK, BRK, ARG, and FRK. The HCK function has been shown to promote renal fibrosis through increasing macrophage proliferation and migration.^39^ However, their role in HSC activation and fibrotic function remains unclear. The PTK activity pattern is the opposite in TGFβ treated condition compared with the results of insulin treatment.

Since the loss of INSR increases HSC growth and cell movement with TGFβ, the *INSR* ^e5‐8^ KO HSCs had hyperactive PTKs that may contribute to these increased HSC cell functions. For instance, FES has been shown to promote cytoskeletal reorganization and cell movement in mast cells.^40^ Although the direct involvement of FES in HSC activation has not been identified, hyperactive FES may enhance the migratory capacity of *INSR* ^e5‐8^ KO HSCs, as observed in our studies. In addition to FES, MER in macrophages has been shown to activate HSCs and induce liver fibrosis through an ERK–TGFβ pathway.^41^ MER has also been demonstrated as a TGFβ‐inducible marker of fibrosis in mouse models of liver, kidney, and lung fibrosis.^42^ Studies on MER indicate it is essential in immune cells and show how MER promotes liver immune cells, like Kupffer cells, to activate HSCs in MASH and hepatic fibrosis.^43^ Detailed mechanisms by which MER regulates HSC activation are yet to be elucidated. Our findings showed that hyperactive MER in *INSR* ^e5‐8^ KO HSCs is correlated with TGFβ‐induced HSC proliferation and movement.

In contrast to PTK's signal initiation function, STKs play essential roles in downstream signal transduction.^44^ A well‐known example of an STK is ERK. It is a signaling hub in many intracellular pathways, including insulin signaling. Our results showed that ERK1, ERK2, ERK5, and ERK7 activities are reduced in *INSR* ^e5‐8^ KO HSCs in response to TGFβ stimulation, compared to scramble control HSC. ERK1 and ERK2 (ERK1/2) are enhancers of HSC proliferation, survival, and resistance to apoptosis and are considered activators for type I collagen synthesis.^45^ Since the INSR signaling pathway activates ERK1/2, it is logical that the loss of INSR in HSCs would lead to reduced ERK1/2 activity, as shown in our results. Although direct regulatory effects of ERK5 on liver fibrosis have not been reported, ERK5's fibrosis‐promoting effects have been demonstrated in the lung.^46^ For ERK7, the regulation of ERK7 in HSC activation remains unexplored. Generally, ERKs tend to enhance HSC activation in response to TGFβ. However, our findings also revealed an unexpected increase in HSC proliferation and migration, suggesting that other signaling pathways may compensate for the loss of ERK activity in these HSCs. We also found that the *INSR* ^e5‐8^ KO HSCs affected insulin's and TGFβ's effects on STK activity. PTKs are primarily upstream regulators in a pathway, whereas STKs are usually downstream messengers of the middle stream cascades.

In addition to these kinase pathways, the top 100 changed pathways found in our RNA‐seq analysis included the collagen‐activated signaling pathway, smooth muscle cell differentiation pathway, SMAD signaling, notch signaling, TGFβ receptor binding, collagen metabolic process, integrin‐mediated cell adhesion, and cell‐matrix adhesion mediator activity. These indicate that INSR is essential in regulating the middle‐stream signaling in responses to insulin and TGFβ stimulations that also drive transcription factor control of gene transcription. This could impact treatments in diabetic patients, as Yen et al. published that patients with insulin‐resistant diabetes who also had liver cirrhosis and used insulin injections were associated with higher risks of death and liver‐related complications than patients with the same conditions but did not use insulin.^47^ MASLD patients typically experience reduced plasma bilirubin levels (hypobilirubinemia), a typical liver disease biomarker^10^, ^48^, ^49^; the decreased levels could be related to its fat‐reducing capabilities,^49^, ^50^ and lowering levels allow lipids to accumulate. Insulin sensitizers improve liver dysfunction associated with MASLD.^48^ Interestingly, bilirubin nanoparticles reduced liver fibrosis in animal models and human LX2 HSCs and lowered ALT and AST liver dysfunction biomarkers.^51^ Bilirubin nanoparticle treatment in obese mice with MASLD suppressed fat accumulation, hepatic inflammation, and lowered AST levels.^52^, ^53^, ^54^ Reducing levels of hepatic UGT1A1 that conjugate bilirubin increases plasma levels.^55^ Inhibiting UGT1A1 may be useful for insulin sensitizing the liver, as it has been demonstrated that an RNAi targeted to the liver increased plasma bilirubin, significantly improved MASLD and insulin sensitivity, and lowered blood glucose and insulin levels in obese mice.^22^ Plasma bilirubin levels have been directly linked to insulin sensitivity, and reduced levels in humans are associated with higher HOMA‐IR and insulin resistance.^56^ Our findings can guide future studies on INSR and HSC function and how they affect MASH and end‐stage liver fibrosis in cirrhotic patients, who usually experience high bilirubin levels.

In conclusion, this study demonstrates the pathways involved in INSR‐mediated HSC activation under insulin or TGFβ stimulation and advocates INSR's effects on HSC activation. There is currently no literature on this topic specifically showing the changes in transcriptional patterns or kinase activities. Our studies reveal multiple potential targets that can guide future studies and contribute to developing better and safer treatment strategies for MASH‐induced liver fibrosis as well as more advanced liver diseases such as cirrhosis. Our study may play an essential role in better understanding HSC activation and liver fibrosis and possibly open new therapeutic targets, as no treatments currently reverse hepatic fibrosis.

## AUTHOR CONTRIBUTIONS

Wang‐Hsin Lee and Terry D. Hinds Jr. conceived and designed the research concepts. Wang‐Hsin Lee, Genesee J. Martinez, Sally N. Pauss, and Zachary A. Kipp performed experiments. Wang‐Hsin Lee performed and analyzed the Real‐time PCR and western blot data. Wang‐Hsin Lee and Cheavar A. Blair performed and analyzed the imaging data. Evelyn A. Bates analyzed RNA sequencing data and performed the pathways analysis. Zachary A. Kipp and Evelyn A. Bates performed the bioinformatic analysis and graphed the kinome data. All authors prepared the manuscript; all authors edited and revised the manuscript. All authors approved the final version of the manuscript.

## FUNDING INFORMATION

This work was not supported by grant funding. However, grants from the National Institutes of Health (NIH), R01DK121797 (T.D.H.J.), R01DA058933 (T.D.H.J.), F31HL170972 (Z.A.K.), F31HL175979 (E.A.B.), and 25PRE1374495 (G.J.M.) supported some author salaries during the studies.

## DISCLOSURES

Nothing to report.

## Supporting information


Figure S1.

## Data Availability

The data for the Hinds Lab Kinome Reports for STK and PTK, KRSA raw data, and spreadsheets containing the Log2 fold change of the family and individual kinases are openly available on FigShare at DOI: 10.6084/m9.figshare.28485599. The RNA sequencing data is available on Sequence Read Archive at BioProject ID: PRJNA1228036.
