# Supplementary material for: Insulin receptor responsiveness governs TGFβ‐induced hepatic stellate cell activation: Insulin resistance instigates liver fibrosis
Source: FASEB J. 2025 Mar 1;39(5):e70427. doi: 10.1096/fj.202402169R (PMC11871568; doi:10.1096/fj.202402169R)
Supplement: Supplementary file 1 — Figure S1. [file FSB2-39-e70427-s001.docx]

**Insulin Receptor Responsiveness Governs TGFβ-induced Hepatic Stellate Cell Activation: Insulin Resistance Instigates Liver Fibrosis**

Wang-Hsin Lee^1,2^, Evelyn A. Bates^1,2^, Zachary A. Kipp^1,2^, Sally N. Pauss^1,2^, Genesee J. Martinez^1,2^, Cheavar A. Blair^1,3,4^, and Terry D. Hinds, Jr^1,2,3,5^

**SUPPLEMENTAL DATA**

**Supplemental Figure 1.** (A) Real-time PCR of *TP53* mRNA expression results in *INSR*^e5-8^ KO and scramble HSCs treated with TGFβ 5 ng/ml for 24 hours. [*, p,0.05; **, p,0.01; n = 3 each; Two-way ANOVA; ± S.E.M.]. (B) Cell cycle measurements using propidium iodide staining in *INSR*^e5-8^ KO and scramble HSCs treated with TGFβ 5 ng/ml for 24 hours. [**, p,0.01; ***, p,0.001, ****, p,0.0001, n = 6 each; Two-way ANOVA; ± S.E.M.].
